# Clinico-Biological Features and Clonal Hematopoiesis in Patients with Severe COVID-19

**DOI:** 10.3390/cancers12071992

**Published:** 2020-07-21

**Authors:** Nicolas Duployez, Jordane Demonchy, Céline Berthon, Julien Goutay, Morgan Caplan, Anne-Sophie Moreau, Anne Bignon, Alice Marceau-Renaut, Delphine Garrigue, Imelda Raczkiewicz, Sandrine Geffroy, Maxime Bucci, Kazali Alidjinou, Julie Demaret, Myriam Labalette, Thierry Brousseau, Annabelle Dupont, Antoine Rauch, Julien Poissy, Sophie Susen, Claude Preudhomme, Bruno Quesnel, on behalf of the Lille Covid Research Network (LICORNE)

**Affiliations:** 1 UMR 9020–UMR-S 1277–Canther–Cancer Heterogeneity, Plasticity and Resistance to Therapies, Institut de Recherche contre le Cancer de Lille, University Lille, CNRS, Inserm, CHU Lille, F-59000 Lille, France; jordane.demonchy@gmail.com (J.D.); celine.berthon@chru-lille.fr (C.B.); alice.marceau@chru-lille.fr (A.M.-R.); imelda.raczkiewicz.etu@univ-lille.fr (I.R.); claude.preudhomme@chru-lille.fr (C.P.); bruno.quesnel@chru-lille.fr (B.Q.); 2 Department of Hematology, CHU Lille, F-59000 Lille, France; sandrine.vandermeersch@chru-lille.fr (S.G.); maxime.bucci@chru-lille.fr (M.B.); 3 Department of Intensive Care, CHU Lille, F-59000 Lille, France; julien.goutay@chru-lille.fr (J.G.); morgan.caplan@chru-lille.fr (M.C.); annesophie.moreau@chru-lille.fr (A.-S.M.); julien.poissy@chru-lille.fr (J.P.); 4 Department of Anesthesia and Critical Care, CHU Lille, F-59000 Lille, France; anne.bignon@chru-lille.fr (A.B.); delphine.garrigue@chru-lille.fr (D.G.); 5 Department of Emergency, CHU Lille, F-59000 Lille, France; 6 Department of Virology, CHU Lille, F-59000 Lille, France; enagnonkazali.alidjinou@chru-lille.fr; 7 Department of Immunology, CHU Lille, F-59000 Lille, France; julie.demaret@chru-lille.fr (J.D.); myriam.labalette@chru-lille.fr (M.L.); 8 Department of Biochemistry, CHU Lille, F-59000 Lille, France; thierry.brousseau@chru-lille.fr; 9 UMR1011-EGID, Pasteur Institute of Lille, University Lille, Inserm, CHU Lille, F-59000 Lille, France; annabelle.dupont@chru-lille.fr (A.D.); antoine.rauch@chru-lille.fr (A.R.); sophie.susen@chru-lille.fr (S.S.); 10 Department of Hemostasis, CHU Lille, F-59000 Lille, France

**Keywords:** SARS-CoV-2, COVID-19, clonal hematopoiesis, CHIP, sequencing, *DNMT3A*, *TET2*

## Abstract

Advanced age or preexisting comorbidities have been characterized as risk factors for severe coronavirus disease 2019 (COVID-19) cases requiring hospitalization and intensive care. In recent years, clonal hematopoiesis (CH) of indeterminate potential (CHIP) has emerged as a risk factor for chronic inflammatory background and subsequent aging-associated diseases. The purpose of this study was to identify biological factors (particularly leukocyte subtypes and inflammatory markers) associated with a risk of clinical deterioration (i.e., orotracheal intubation (OTI)) and to determine whether CH was likely to influence clinical and biological behavior in patients with severe COVID-19 requiring hospitalization. Here, we describe clinical and biological features, including the screening of CHIP mutants in a well-annotated cohort of 122 hospitalized patients with a laboratory-confirmed diagnosis of COVID-19 (55% requiring OTI). We showed that elevated white blood cell counts, especially neutrophils and high C-reactive protein (CRP) levels at admission, were associated with an increased requirement of OTI. We noticed a high prevalence of CH (25%, 38%, 56%, and 82% of patients aged <60 years, 60–70 years, 70–80 years, and >80 years) compared to a retrospective cohort of patients free of hematological malignancy explored with the same pipelines (10%, 21%, 37%, and 44%). However, the existence of CH did not significantly impact clinical outcome, including OTI or death, and did not correlate with other laboratory findings.

## 1. Introduction

In December 2019, the world faced an outbreak of coronavirus disease 2019 (COVID-19), caused by the severe acute respiratory syndrome coronavirus 2 (SARS-CoV-2). Although a large proportion of affected patients present with little or mild flu-like symptoms, the disease may cause severe or fatal complications in some people. This has led to research efforts to identify people at higher risk of severe illness and death. Advanced age or preexisting diseases, such as hypertension, cardiovascular diseases, obesity, diabetes, chronic respiratory diseases, or cancers, have been characterized as risk factors for severe COVID-19 cases requiring hospitalization and intensive care [1]. Severe complications have been attributed, at least in part, to hyperinflammation and inappropriate cytokine release [2]. Serum profiling of COVID-19 patients has revealed a distinct inflammatory response characterized by high levels of interleukin-6 (IL-6) and reduced type I interferon [3].

In recent years, large-scale sequencing studies have demonstrated that detectable somatic mutations are common in the peripheral blood of healthy individuals, especially at advanced ages [4,5,6,7]. This condition, named clonal hematopoiesis (CH) of indeterminate potential (CHIP), implies that genes recurrently mutate in myeloid hematological malignancies, among which the master epigenetic regulators DNA-methyltransferase 3A (*DNMT3A*) and its partner Tet-methylcytosine dioxygenase 2 (*TET2*) are the most frequently involved [8]. The etiology, biological impact on hematopoiesis, and evolution of individuals with CHIP currently represent notable fields of research. Case control studies have shown that CHIP was associated with a higher risk of hematological cancer [4,5], as expected, but also with an increase in incident coronary heart disease, ischemic stroke and all-cause mortality [4,9,10], and chronic obstructive pulmonary disease [6,7]. Interestingly, further studies have suggested a connection between cytokine-mediated processes and CHIP [6,9,11,12,13,14], with higher serum IL-6 [15] and C-reactive protein (CRP) [16] levels in CHIP carriers. Studies using murine models have also demonstrated that *Dnmt3a-* and *Tet2*-deficient animals were characterized by impaired production of type I interferon and increased IL-6 production, respectively, compared to wild-type mice [12,17].

Considering the putative connection between CHIP and comorbidities or inappropriate inflammatory responses (both of which have been associated with more severe forms of COVID-19), we assumed that patients with severe COVID-19 could be characterized by a distinct CHIP profile. We therefore conducted an observational study of individuals referred to our center during the COVID-19 pandemic. In this report, we describe clinical and biological features, including the screening of CHIP mutants, in a well-annotated cohort of 122 hospitalized patients with a laboratory-confirmed diagnosis of COVID-19 (55% requiring orotracheal intubation (OTI) in the intensive care unit (ICU)) at the Centre Hospitalier Universitaire de Lille (CHU Lille, France).

## 2. Methods

### 2.1. Patients and Samples

All patients with a laboratory-confirmed diagnosis of COVID-19 who were hospitalized at the CHU Lille and had a complete blood count (CBC) performed between 8 April and 23 April 2020 were enrolled in this study (*n* = 122). The CHU Lille is the tertiary care center for Nord-Pas-de-Calais, France (approximately 4 million inhabitants). All patients were positive for SARS-CoV-2 infection, as determined using real-time reverse transcription-polymerase chain reaction (RT-PCR) [18]. Samples used for CBC were stored for DNA extraction. A trained team of physicians prospectively collected and reviewed the epidemiological data, past medical history, treatments, clinical and biological data, and outcomes in these patients [19]. This observational study was based on medical records and was in strict compliance with the French reference methodology MR-004 and approved by the Institutional Data Protection Authority of CHU Lille. This study was approved by the French institutional authority for personal data protection (Commission Nationale de l’Informatique et des Libertés (CNIL), registration number DEC20-086), and ethics comittee (ID-CRB 2020-A00763-36). The protocol was registered as a clinical trial (registration numbers NCT-04327180 and NCT-04341792 for patients admitted to ICU and patients who consulted in the emergency department, respectively).

A retrospective cohort of 376 patients free of hematological malignancy and screened for mutations with the same pipeline before the COVID-19 pandemic was used for comparison. Briefly, samples from 1833 patients were sequenced between January 2019 and January 2020 at the Laboratory of Hematology of CHU Lille (see details of gene panel and pipeline below). Samples obtained prior to January 2019 were not selected because of subtle changes in bioinformatics pipelines that could have affected the sensitivity threshold of the sequencing. Physicians who performed the analyses prospectively registered final diagnoses during this period. Among these patients, 479 (26%) were diagnosed with acute myeloid leukemia, 152 (8%) with myelodysplastic/myeloproliferative neoplasms, 253 (14%) with myeloproliferative neoplasms, and 573 (31%) with myelodysplastic syndromes. The 376 remaining patients (21%) did not meet sufficient criteria for a diagnosis of hematological malignancy and were considered to have ICUS (idiopathic cytopenia of undetermined significance), CCUS (clonal cytopenia of undetermined significance), or CHIP (clonal hematopoiesis of indeterminate potential) and were used for comparison. High-throughput sequencing (HTS) technology and pipelines were identical to those used for COVID-19-positive individuals. This retrospective cohort comprised 185 males and 191 females. Median age was 69 years (range, 50–93). Age groups were distributed as follows: <60 years: *n* = 82 (22%); (60–70 years): *n* = 108 (29%); (70–80 years): *n* = 129 (34%); >80 years: *n* = 57 (15%). 

### 2.2. Molecular Analyses

Genomic DNA extracted from whole blood was studied by HTS of 36 genes recurrently mutated in myeloid malignancies. The studied panel included genes encoding proteins involved in kinase signaling (*CALR* (exon 9), *CBL* (exons 8–9), *CSF3R* (exons 14–18), *FLT3* (exon 20), *JAK2* (exons 12, 14), *KIT* (exons 8–11, 17), *KRAS* (exons 2–3), *MPL* (exon 10), *NRAS* (exons 2–3), *PTPN11* (exons 3, 13), *RIT1* (exon 5), *SETBP1* (exon 4)), transcription factors (*ETV6* (exons 1–8), *GATA2* (exons 2–6), *RUNX1* (exons 1–6)), tumor suppressors (*PHF6* (exons 2–10), *TP53* (exons 3–11), *WT1* (exons 7, 9)), chromatin modifiers (*ASXL1* (exons 11–12), *BCOR* (exons 2–15), *BCORL1* (exons 1–12), *EZH2* (exons 2–20)), DNA methylation (*DNMT3A* (exons 2–23), *IDH1* (exon 4), *IDH2* (exon 4), *TET2* (exons 3–11)), cohesin complex (*NIPBL* (exons 2–47), *RAD21* (exons 2–14), *SMC1A* (exons 1–25), *SMC3* (exons 1–29), *STAG2* (exons 3–35)), RNA splicing (*SF3B1* (exons 13–16), *SRSF2* (exon 1), *U2AF1* (exons 2, 6), *ZRSR2* (exons 1–11)), and *NPM1* (exon 11). Notably, our panel did not include *PPM1D*, for which mutations have been frequently reported in CHIP, especially in patients treated for prior non-hematological cancer. Libraries were prepared using the Ampliseq System, according to the manufacturer’s instructions, and run on Ion S5 (Thermo Fisher, Waltham, MA, USA). Raw data were analyzed with both Torrent Browser (Thermo Fisher) and SeqNext (JSI Medical System, Los Angeles, CA, USA) and visualized with the homemade NGS report software v1 (CHU Lille). Due to technical limitations of the HTS technology on Ion S5, sequencing of the *ASXL1* hotspot (c.1934dupG) was screened by fragment analysis and subsequent Sanger sequencing in all samples. Thus, the sensitivity threshold for this mutational hotspot was supposed to be 10% of the variant allele frequency (VAF). 

Most reports use a VAF of at least 2% to define CHIP, which represents the sensitivity threshold of current HTS technologies used in the clinical setting [20]. Sequencing data variability and threshold of our HTS technology were determined by serial sequencing of the multiplex NGS Tru-Q DNA 7 (Horizon Diagnostics, Cambridge, UK). In our hands, the measured VAF for a variant with an expected VAF of 2% will be between 1.1% and 2.9%. A high depth of coverage (>1500 X) was obtained for all CHIP-associated genes [4] in this panel (Figure S1) and mutations were detected until a filtered VAF of 1.5%–2% was obtained, with at least 20 reads carrying the variant (Table S1). The median depth of coverage was 3720 X and 2629 X for *DNMT3A* and *TET2* full genes, respectively. 

Variant interpretation was performed considering minor allele frequencies (MAF) in the public GnomAD database of polymorphisms (variants with MAF > 0.02 in overall population/global ancestry or sub-continental ancestry were excluded), prevalence, and interpretation in our in-house database of more than 8000 samples validated for a clinical purpose and VAF compatibility with a somatic state. Additional in silico predictions were performed when possible. Frameshift and nonsense variants were always considered as relevant mutations. Single nucleotide variant effects on protein function were predicted using SIFT (Sorting Tolerant from Intolerant) and PolyPhen-2. The effects of splicing variants were predicted with Human Splicing Finder version 3.1. Additional criteria for the classification of variants as somatic driver mutations are given in Table S2. The exclusion of sequencing artifacts was performed by estimating the sequencing noise at identified variants’ positions in other DNA samples. No variants identified in the present study were found in 65 serial sequencings of a commercial genomic negative DNA (CytoScan™ Reagent Kit Components, Thermofisher, Waltham, MA, USA). Since genetic background and polymorphisms could give rise to some artifacts, we also ensured that no identified variants were found more than expected in a series of 4240 DNA samples from patients suspected of myeloid malignancies (Table S3). Finally, in 9 patients with identified variants (12 different somatic mutations), we repeated the sequencing in another sample (DNA sampled on the day of admission, i.e., between 7 and 16 days before the first analysis) and confirmed the results (Figure S2).

### 2.3. Laboratory Blood Dosages and CBC

Laboratory blood dosages and CBC were prospectively assessed by standard methods as part of patient care at the Biology and Pathology Center (CHU Lille). Serum IL-6 levels were determined using a human IL-6 BD OptEIA enzyme-linked immunosorbent assay (ELISA) (BD Biosciences, San Diego, CA, USA), according to the manufacturer’s instructions. This assay had a range of 2.5–300 pg/mL.

### 2.4. Statistical Analyses

The purpose of this study was to identify biological factors (particularly leukocyte subtypes and inflammatory markers) associated with a risk of clinical deterioration (i.e., OTI) and to determine whether CH was likely to influence clinical and biological behavior in patients with severe COVID-19 requiring hospitalization. Hospitalization in the ICU per se was not considered appropriate since the criteria for admission changed during the pandemic. Total CH rate, *DNMT3A*, and *TET2* mutations were tested successively. The Mann–Whitney U test or t-test (continuous variables) and chi-square test or Fisher’s exact test (categorical variables) were used for comparisons between groups. Binary logistic regression was used to determine odds ratios (ORs). A 2-sided *p*-value of 0.05 or less was considered to be statistically significant. All statistical tests were performed with the SPSS 22.0 (IBM Corp., Armonk, NY, USA) software package. 

## 3. Results

**Figure 1:**
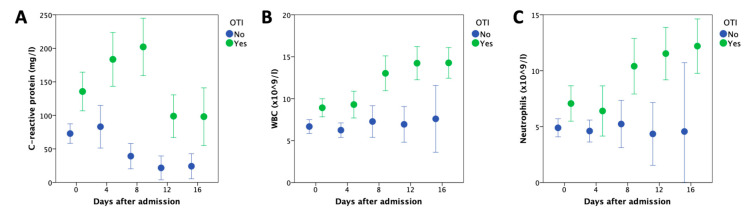
Kinetics of laboratory values (mean (95% CI)) during hospitalization in COVID-19 patients according to the requirement (green) or not (blue) of orotracheal intubation (OTI). (**A**) C-reactive protein; (**B**) white blood cell count (WBC); (**C**) neutrophils.

The cohort of hospitalized COVID-19 patients included 93 males and 29 females. The median age was 65 years (range, 22–95). Among these patients, 89 (73%) were transferred to the ICU during hospitalization and 67 (55%) required OTI. Twenty-two (18%) and six (5%) patients experienced venous thromboembolic disease (pulmonary embolism, *n* = 18; deep vein thrombosis, *n* = 4) and arterial disease (strokes, *n* = 4; myocardial infarction, *n* = 2), respectively. At the time of this report, 95 patients were cured (defined by discharge from the hospital), 17 died (mortality rate: 15%), and 10 were still hospitalized. High levels of white blood cell count (WBC), especially neutrophils and C-reactive protein, were associated with more severe forms, defined by an increased requirement of OTI (Figure 1). ORs for OTI were 3.647 (95% CI: 1.264–10.529, *p* = 0.017), 1.196 (95% CI: 1.054–1.358, *p* = 0.006), and 1.286 (95% CI: 1.053–1.570, *p* = 0.013) for logCRP, WBC, and neutrophil counts (considered as continuous variables), respectively. Other characteristics of COVID-19 patients are reported in Table 1. In the absence of samples collected prior to the COVID-19 outbreak, we assumed that the observed biological data were the result of both the patient’s previous condition and infectious disease.

**Figure 2:**
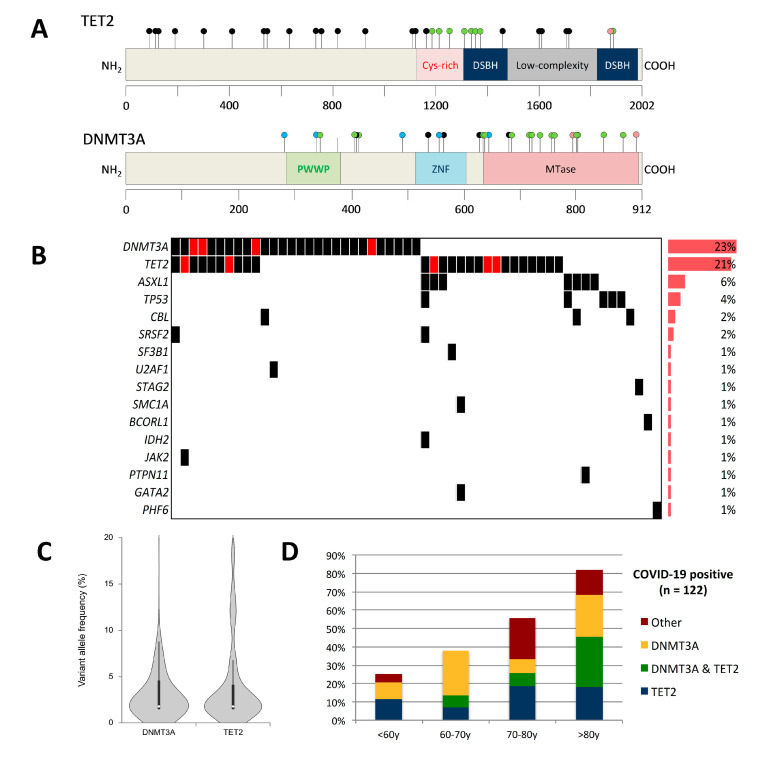
CH in COVID-19 patients requiring hospitalization. (**A**) Lollipop plots depicting *TET2* and *DNMT3A* mutations in COVID-19 patients. Black, green, pink, and blue dots indicate frameshift/nonsense, missense, in frame, and splicing mutations, respectively. (**B**) Molecular landscape showing co-mutations in 55 clonal hematopoiesis-positive patients. Red boxes indicate several mutations within the same gene. (**C**) Violin plot showing the distributions of variant allele frequencies for *TET2* and *DNMT3A* mutations. (**D**) Frequency of CH among age groups in COVID-19 patients.

A total of 91 mutations were found in 55 individuals (45% of the whole cohort) (Figure 2A,B, Table S4). *DNMT3A* and *TET2* mutations were by far the most common, concerning 28 (23%) and 26 patients (21%), respectively. Overall, mutations in *DNMT3A* and/or *TET2* were present in 36% (44/122) of COVID-19 patients and were found in 80% (44/55) of those with CH. Ten patients had both *DNMT3A* and *TET2* mutations. Other recurrently mutated genes included *ASXL1* (*n* = 7; 6%) and *TP53* (*n* = 5; 4%). The average number of detected mutations among patients with CH was one (range, 1–5). The median variant allele frequency (VAF) was 2% (Figure 2C), but 10 patients had mutations with VAFs higher than 10% (none of them were known to have a hematological malignancy). Particular attention was paid to other CBCs during management, especially for the existence of monocytosis, blasts or evolution of cytopenias. None of them had additional argument for an active malignancy. As expected, CH was more frequent in elderly people, and its prevalence increased with age (Figure 2D). CH affected 25%, 38%, 56%, and 82% of patients aged <60 years, 60–70 years, 70–80 years, and >80 years, respectively. The median age of CH-positive and CH-negative patients was 72 years and 60 years, respectively (*p* < 0.001). Laboratory values from CBC and inflammatory markers did not differ significantly between CH-positive and CH-negative patients (Table 1, Figure S3). Serum levels of IL-6 in patients with available material were not different between CH-positive vs. CH-negative, *DNMT3A*-positive vs. *DNMT3A*-negative, and *TET2*-positive vs. *TET2*-negative patients. Data about other cytokine levels were not available. Univariate analyses showed no significant difference in outcome, clinical symptoms, or comorbidities between the two groups, except for myocardial infarction and obliterative arterial disease, which were reported more frequently in the medical history of CH-positive patients (*p* = 0.046). Intriguingly, OTI requirement was less frequent in patients with *DNMT3A* mutations (10/28 (36%) vs. 57/94 (61%) in mutated vs. unmutated; OR = 0.361, 95% CI: 0.150–0.867, *p* = 0.023) but the small size of our cohort and the absence of a validation cohort do not allow for further speculations. This was not observed for *TET2* mutant carriers (12/26 (46%) vs. 55/96 (57%) in mutated vs. unmutated; OR = 0.639, 95% CI: 0.268–1.526, *p* = 0.313).

**Figure 3:**
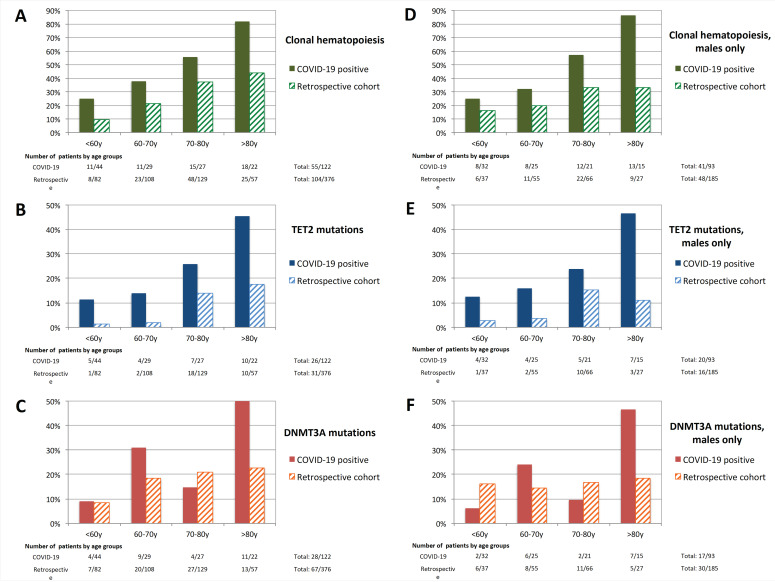
Frequency of individuals with (**A**,**D**) clonal hematopoiesis, (**B**,**E**) *TET2* mutations, and (**C**,**F**) *DNMT3A* mutations in COVID-19 positive patients and patients from the retrospective cohort. Subfigures (**D**–**F**) show frequency for males only.

We then compared the frequency of CH in hospitalized COVID-19 patients with a retrospective cohort of 376 patients free of hematological malignancy sequenced with the same pipelines before the COVID-19 pandemic. CH prevalence among age groups appeared higher in COVID-19 patients compared to patients from this cohort (10%, 21%, 37%, and 44% of patients aged <60 years, 60–70 years, 70–80 years, and >80 years, respectively; Figure 3), especially due to a higher rate of *TET2* mutations in COVID-19 patients. Since the cohort of COVID-19-patients was male biased, comparisons were also performed in males only, showing the same trends. Notably, there was no specific pattern of mutation according to gender in the retrospective cohort (Table S5).

After adjustment for age, the prevalence OR of CH was 3.182 (95% CI: 1.944–5.209, *p* < 0.001) in COVID-19 patients. The same analyses were performed for *DNMT3A* (presence vs. absence) and *TET2* mutations (presence vs. absence) and showed prevalence ORs of 1.735 (95% CI: 1.000–3.010, *p* = 0.050) and 3.940 (95% CI: 2.095–7.410, *p* < 0.001), respectively. 

## 4. Discussion

In this report, we describe the clinical and biological findings, including the extensive screening of CH in hospitalized COVID-19 patients, of our institution. We confirmed high CRP levels and WBC/neutrophil counts as biological predictors of OTI requirement in patients with severe COVID-19 requiring hospitalization, in line with previous reports [21].

Our results show a higher rate of CH, especially *TET2* mutations, in both COVID-19 patients and people from the retrospective cohort compared to data from the literature [4,5]. However, it is important to note that different panels, sequencing technologies, or bioinformatics pipelines may introduce biases in the sensitivity threshold of CH detection, thereby greatly limiting such comparisons. This may in part be related to a broader *TET2* coverage in the present study. Additionally, it should be noted that certain regions, including the *ASXL1* hotspot, are prone to technical artifacts with IonTorrent sequencing platforms, which could have limited *ASXL1* mutant detection in our study. Finally, individuals in our retrospective cohort should not be considered as healthy people since most of them had unexplained cytopenia. However, this would likely increase the prevalence of CH in our control population and therefore not affect the conclusions regarding the higher CH frequency in COVID-19 patients. We acknowledge that this finding may suffer from several biases, including the absence of sampling prior to COVID-19, the relatively small size of the studied group, and the absence of matched controls for confounding factors including comorbidities. Notably, the impact of acute inflammatory states on CH (possibly affecting clone selection or expansion) in COVID-19 patients remains unknown. Indeed, it has been suggested that the emergence of CH (especially *TET2* mutants) may be facilitated in response to inflammatory stress [22,23]. Given the lack of samples collected prior to COVID-19 infection and in non-hospitalized patients, we were not able to determine whether the high frequency of CH in these patients could be associated with a higher rate of hospitalization for COVID-19 or reflect a consequence of the acute inflammatory state. Additionally, although all biological and clinical were prospectively collected and reviewed, we could not exclude the existence of other undiagnosed pathological conditions in our patients. Overall, we show that CH does not significantly impact clinical and biological findings in COVID-19 patients or outcomes, including OTI or death. Given that 75% of CH variants were identified with a VAF below 5%, it was expected that this would have little or minimal clinical consequence. Due to the low number of individuals presenting with larger clones, we were not able to perform analyses regarding higher VAF cutoffs.

## 5. Conclusions

In conclusion, we describe clinical and biological findings in patients with severe COVID-19 requiring hospitalization. Elevated white blood cell counts, especially neutrophils and high CRP levels at admission, were associated with an increased requirement of OTI. We also report a high frequency of CH in this population, with a lack of impact on clinico-biological findings and outcome. A longer follow-up period will be necessary to estimate the long-term consequences of CH on the outcome of patients, including the occurrence of hematological malignancies or aging-associated comorbidities.

## Figures and Tables

**Table 1:** Clinical and biological characteristics of COVID-19 patients.

Characteristics	All COVID-19 Patients	Clonal Hematopoiesis-Negative	Clonal Hematopoiesis-Positive	*p*-Value
Number of patients	122	67	55	
Male/Female, sex ratio (%)	93/29 (76%)	52/15 (78%)	41/14 (75%)	0.831
Median age, years (range)	65 (22–95)	60.2 (22.2–87.3)	71.7 (29.5–94.6)	<0.001
<60 years	44 (36%)	33 (49%)	11 (20%)	<0.001
(60–70 years)	29 (24%)	18 (27%)	11 (20%)	
(70–80 years)	27 (22%)	12 (18%)	15 (27%)	
>80 years	22 (18%)	4 (6%)	18 (33%)	
Body-mass index, kg/m^2^ (median (range))	28.9 (15.1–53.3)	29.8 (16.6–53.3)	28 (15.1–41.9)	0.249
Medical history (*n* (%))				
Smoking	21 (17%)	10 (15%)	11 (20%)	0.480
Diabete	34 (28%)	18 (27%)	16 (29%)	0.841
Respiratory illness	30 (25%)	17 (25%)	13 (24%)	1.000
Cardiac failure	17 (14%)	8 (12%)	9 (16%)	0.601
Arterial hypertension	65 (53%)	31 (46%)	34 (62%)	0.102
Stroke	10 (8%)	3 (4%)	7 (13%)	0.183
Myocardial infarction, obliterative arterial disease	14 (11%)	4 (6%)	10 (18%)	0.046
Cirrhosis	3 (2%)	2 (3%)	1 (2%)	1.000
Renal insufficiency	11 (9%)	5 (7%)	6 (11%)	0.541
Hematological malignancy	6 (5%)	4 (6%)	2 (4%)	0.689
Solid tumor	10 (8%)	3 (4%)	7 (13%)	0.183
Immunodepression	9 (7%)	7 (10%)	2 (4%)	0.183
Simplified Acute Physiology Score (SAPS II) *, median (range)	40 (12–83)	39 (12–83)	41 (21–81)	0.807
Symptoms at admission (*n* (%))				
Fever	101 (83%)	57 (85%)	44 (80%)	0.480
Cough	82 (67%)	43 (64%)	39 (71%)	0.446
Expectorations	14 (11%)	7 (10%)	7 (13%)	0.779
Dyspnea	111 (91%)	64 (96%)	47 (85%)	0.064
Headache	7 (6%)	5 (8%)	2 (4%)	0.453
Tiredness	80 (66%)	46 (69%)	34 (62%)	0.450
Muscle pain	28 (23%)	16 (24%)	12 (22%)	0.832
Gastrointestinal symptoms	34 (28%)	25 (37%)	9 (16%)	0.014
Otolaryngeal symptoms	16 (13%)	7 (10%)	9 (16%)	0.422
Measures at admission (median (range))				
Heart rate (bpm)	94 (50–147)	93.5 (50–141)	95.5 (59–147)	0.756
Systolic blood pressure (mm Hg)	124 (58–200)	127 (80–200)	118 (58–160)	0.111
Diastolic blood pressure (mm Hg)	66.5 (40–124)	67.5 (41–124)	66 (40–124)	0.751
Mean blood pressure (mm Hg)	83 (51–141)	84.5 (55–141)	81.5 (51–133)	0.722
Body temperature (°C)	37.7 (34.5–40.6)	37.7 (35.5–40.6)	37.6 (34.5–39.7)	0.525
Respiratory rate (rpm)	24 (10–47)	24 (13–51)	23 (10–47)	0.819
Laboratory findings (median (range))				
White blood cell count (number × 10^9^/L)	8.1 (2–28.2)	8.8 (2–28.2)	7.8 (2.5–26.8)	0.762
Neutrophils (number × 10^9^/L)	6.2 (0.9–24.3)	6.4 (0.9–24.3)	5.6 (1.5–22.5)	0.931
Lymphocytes (number × 10^9^/L)	1 (0–4.2)	1.2 (0–4.2)	1 (0.3–2.4)	0.254
Monocytes (number × 10^9^/L)	0.6 (0–1.5)	0.6 (0–1.3)	0.7 (0–1.5)	0.801
Red blood cell count (number × 10^12^/L)	3.5 (1.9–5.1)	3.4 (1.9–5.1)	3.6 (2.4–5.1)	0.175
Hemoglobin concentration (g/L)	10.3 (5.7-15.1)	10.2 (5.7–15.1)	10.4 (7.1–14.4)	0.291
Hematocrit (%)	31.7 (18.2–44.5)	31.6 (18.2–44)	32.6 (22.4–44.5)	0.184
Mean cell volum (fl)	90.3 (80–107.5)	90.6 (80–107.5)	89.6 (80.5–99)	0.350
Mean cell hemoglobin (pg)	29.6 (22.5–37.2)	29.8 (24.8–37.2)	29.4 (22.5–33.6)	0.277
Mean cell hemoglobin concentration (g/L)	32.6 (27.3–36.8)	32.6 (29.5–36.8)	32.6 (27.3–35.3)	0.589
Platelet count (number × 10^9^/L)	294 (18–1006)	296.5 (18–763)	281 (62–1006)	0.220
Mean platelet volume (fl)	10.4 (8–14.5)	10.4 (8.4–14.5)	10.4 (8–12.4)	0.445
C-reactive protein (mg/L)	57 (2–345)	61 (2–322)	55 (6–345)	0.284
Procalcitonin (µg/L)	0.2 (0.1–188)	0.2 (0.1–188)	0.2 (0.1–16)	0.194
Ferritin (µg/L)	962 (69–9900)	1009.5 (140–9900)	814 (69–2637)	0.360
Fibrinogen (g/L)	6.5 (1.6–10.3)	6.6 (2.5–10.3)	6.1 (1.6–9.7)	0.153
Interleukine-6 (ng/L)	38.6 (2.5–≥ 300)	32.8 (2.5–300)	44.2 (4.6–300)	0.365
During hospitalization (*n* (%))				
Admission in reanimation intensive care unit	89 (73%)	56 (84%)	33 (60%)	0.004
Orotracheal intubation	67 (55%)	42 (63%)	25 (45%)	0.069
High-flow nasal cannula oxygenation	37 (30%)	22 (33%)	15 (27%)	0.557
Noninvasive ventilation	43 (35%)	26 (39%)	17 (31%)	0.447
ExtraCorporeal Membrane Oxygenation	17 (14%)	12 (18%)	5 (9%)	0.195
Sympathomimetic amines	59 (48%)	37 (55%)	22 (40%)	0.105
Extrarenal epuration	21 (17%)	15 (22%)	6 (11%)	0.147
Use of corticosteroids	54 (44%)	32 (48%)	22 (40%)	0.465
Outcome (*n* (%))				
Venous thromboembolic disease	22 (18%)	12 (18%)	10 (18%)	1.000
Arterial disease	6 (5%)	3 (4%)	3 (5%)	1.000
Death †	17 (15%)	10 (17%)	7 (13%)	0.793

* Determined only at admission to the ICU. † At time of this report, 10 patients were still hospitalized and were excluded from the measurement of mortality rates.

## Supplementary Materials

The following are available online at https://www.mdpi.com/2072-6694/12/7/1992/s1. Figure S1: Median depth of sequencing coverage per gene, Figure S2: Repeated sequencing in 9 CH-positive patients, Figure S3: Kinetics of laboratory values during hospitalization in COVID-19 patients according to the presence (red boxes) or absence (blue boxes) of CH, Table S1: Determination of the threshold of detection of the high-throughput sequencing assay, Table S2: Target genes and driver classification, Table S3: Variant calling of identified variants in other DNA samples sequenced in the same library (40 DNA samples in each library) and other libraries (4240 DNA samples with the same panel), Table S4: Clonal hematopoiesis-associated variants identified in hospitalized COVID-19-positive patients (*n* = 122), Table S5: Frequency of clonal hematopoiesis, *DNMT3A* mutations, and *TET2* mutations among age groups in the retrospective cohort according to gender.

## Data Sharing

All sequence data, BAM files, and VCF files are available on demand to the corresponding author. Clinical and biological data of COVID-19 patients are available to other researchers upon request.

## Abbreviations

CBC	Complete blood count
CH	Clonal hematopoiesis
CHIP	Clonal hematopoiesis of indeterminate potential
CI	Confidence interval
COVID-19	Coronavirus disease 2019
CRP	C-reactive protein
HTS	High-throughput sequencing
ICU	Intensive care unit
IL-6	Interleukin-6
OR	Odds ratio
OTI	Orotracheal intubation
SARS-CoV-2	Severe acute respiratory syndrome coronavirus 2
VAF	Variant allele frequency
WBC	White blood cell count

## References

[1] Simonnet A., Chetboun M., Poissy J., Raverdy V., Noulette J., Duhamel A., Labreuche J., Mathieu D., Pattou F., Jourdain M. (2020). High Prevalence of Obesity in Severe Acute Respiratory Syndrome Coronavirus-2 (SARS-CoV-2) Requiring Invasive Mechanical Ventilation. Obesity.

[2] Chen G., Wu D., Guo W., Cao Y., Huang D., Wang H., Wang T., Zhang X., Chen H., Yu H. (2020). Clinical and immunological features of severe and moderate coronavirus disease 2019. J. Clin. Investig..

[3] Blanco-Melo D., Nilsson-Payant B.E., Liu W.-C., Uhl S., Hoagland D., Møller R., Jordan T.X., Oishi K., Panis M., Sachs D. (2020). Imbalanced Host Response to SARS-CoV-2 Drives Development of COVID-19. Cell.

[4] Jaiswal S., Fontanillas P., Flannick J., Manning A., Grauman P.V., Mar B.G., Lindsley R.C., Mermel C.H., Burtt N., Chavez A. (2014). Age-Related Clonal Hematopoiesis Associated with Adverse Outcomes. N. Engl. J. Med..

[5] Genovese G., Kähler A.K., Handsaker R.E., Lindberg J., Rose S.A., Bakhoum S.F., Chambert K., Mick E., Neale B.M., Fromer M. (2014). Clonal Hematopoiesis and Blood-Cancer Risk Inferred from Blood DNA Sequence. N. Engl. J. Med..

[6] Buscarlet M., Provost S., Zada Y.F., Barhdadi A., Bourgoin V., Lépine G., Mollica L., Szuber N., Dubé M.-P., Busque L. (2017). *DNMT3A* and *TET2* dominate clonal hematopoiesis and demonstrate benign phenotypes and different genetic predispositions. Blood.

[7] Zink F., Stacey S.N., Norddahl G.L., Frigge M.L., Magnusson O.T., Jonsdottir I., Thorgeirsson T.E., Sigurdsson A., Gudjonsson S.A., Gudmundsson J. (2017). Clonal hematopoiesis, with and without candidate driver mutations, is common in the elderly. Blood.

[8] López-Moyado I.F., Rao A. (2020). DNMT3A and TET2 mutations reshape hematopoiesis in opposing ways. Nat. Genet..

[9] Jaiswal S., Natarajan P., Silver A.J., Gibson C.J., Bick A.G., Shvartz E., McConkey M., Gupta N., Gabriel S., Ardissino D. (2017). Clonal Hematopoiesis and Risk of Atherosclerotic Cardiovascular Disease. N. Engl. J. Med..

[10] Dorsheimer L., Assmus B., Rasper T., Ortmann C.A., Ecke A., Abou-El-Ardat K., Schmid T., Brüne B., Wagner S., Serve H. (2019). Association of Mutations Contributing to Clonal Hematopoiesis with Prognosis in Chronic Ischemic Heart Failure. JAMA Cardiol..

[11] Sano S., Oshima K., Wang Y., Katanasaka Y., Sano M., Walsh K. (2018). CRISPR-Mediated Gene Editing to Assess the Roles of Tet2 and Dnmt3a in Clonal Hematopoiesis and Cardiovascular Disease. Circ. Res..

[12] Zhang Q., Zhao K., Shen Q., Han Y., Gu Y., Li X., Zhao D., Liu Y., Wang C., Zhang X. (2015). Tet2 is required to resolve inflammation by recruiting Hdac2 to specifically repress IL-6. Nature.

[13] Sano S., Oshima K., Wang Y., MacLauchlan S., Katanasaka Y., Sano M., Zuriaga M.A., Yoshiyama M., Goukassian D., Cooper M.A. (2018). Tet2-Mediated Clonal Hematopoiesis Accelerates Heart Failure Through a Mechanism Involving the IL-1β/NLRP3 Inflammasome. J. Am. Coll. Cardiol..

[14] Cull A.H., Snetsinger B., Buckstein R., Wells R.A., Rauh M.J. (2017). Tet2 restrains inflammatory gene expression in macrophages. Exp. Hematol..

[15] Cook E.K., Izukawa T., Young S., Rosen G., Jamali M., Zhang L., Johnson D., Bain E., Hilland J., Ferrone C.K. (2019). Comorbid and inflammatory characteristics of genetic subtypes of clonal hematopoiesis. Blood Adv..

[16] Busque L., Sun M., Buscarlet M., Ayachi S., Feroz Zada Y., Provost S., Bourgoin V., Mollica L., Meisel M., Hinterleitner R. (2020). High-sensitivity C-reactive protein is associated with clonal hematopoiesis of indeterminate potential. Blood Adv..

[17] Li X., Zhang Q., Ding Y., Liu Y., Zhao D., Zhao K., Shen Q., Liu X., Zhu X., Li N. (2016). Methyltransferase Dnmt3a upregulates HDAC9 to deacetylate the kinase TBK1 for activation of antiviral innate immunity. Nat. Immunol..

[18] Huang C., Wang Y., Li X., Ren L., Zhao J., Hu Y., Zhang L., Fan G., Xu J., Gu X. (2020). Clinical features of patients infected with 2019 novel coronavirus in Wuhan, China. Lancet.

[19] Poissy J., Goutay J., Caplan M., Parmentier E., Duburcq T., Lassalle F., Jeanpierre E., Rauch A., Labreuche J., Susen S. (2020). Pulmonary Embolism in COVID-19 Patients: Awareness of an Increased Prevalence. Circulation.

[20] Cook E.K., Luo M., Rauh M.J. (2020). Clonal hematopoiesis and inflammation: Partners in leukemogenesis and comorbidity. Exp. Hematol..

[21] Terpos E., Ntanasis-Stathopoulos I., Elalamy I., Kastritis E., Sergentanis T.N., Politou M., Psaltopoulou T., Gerotziafas G., Dimopoulos M.A. (2020). Hematological findings and complications of COVID-19. Am. J. Hematol..

[22] Abegunde S.O., Buckstein R., Wells R.A., Rauh M.J. (2018). An inflammatory environment containing TNFα favors Tet2-mutant clonal hematopoiesis. Exp. Hematol..

[23] Cai Z., Kotzin J.J., Ramdas B., Chen S., Nelanuthala S., Palam L.R., Pandey R., Mali R.S., Liu Y., Kelley M.R. (2018). Inhibition of Inflammatory Signaling in Tet2 Mutant Preleukemic Cells Mitigates Stress-Induced Abnormalities and Clonal Hematopoiesis. Cell Stem Cell.

