# Supplementary material for: Clinico-Biological Features and Clonal Hematopoiesis in Patients with Severe COVID-19
Source: Cancers (Basel). 2020 Jul 21;12(7):1992. doi: 10.3390/cancers12071992 (PMC7409316; doi:10.3390/cancers12071992)
Supplement: Supplementary file 1 [file cancers-12-01992-s001.pdf]

Supplementary Materials:

Clinico-Biological Features and Clonal Hematopoiesis in Patients with Severe COVID-19

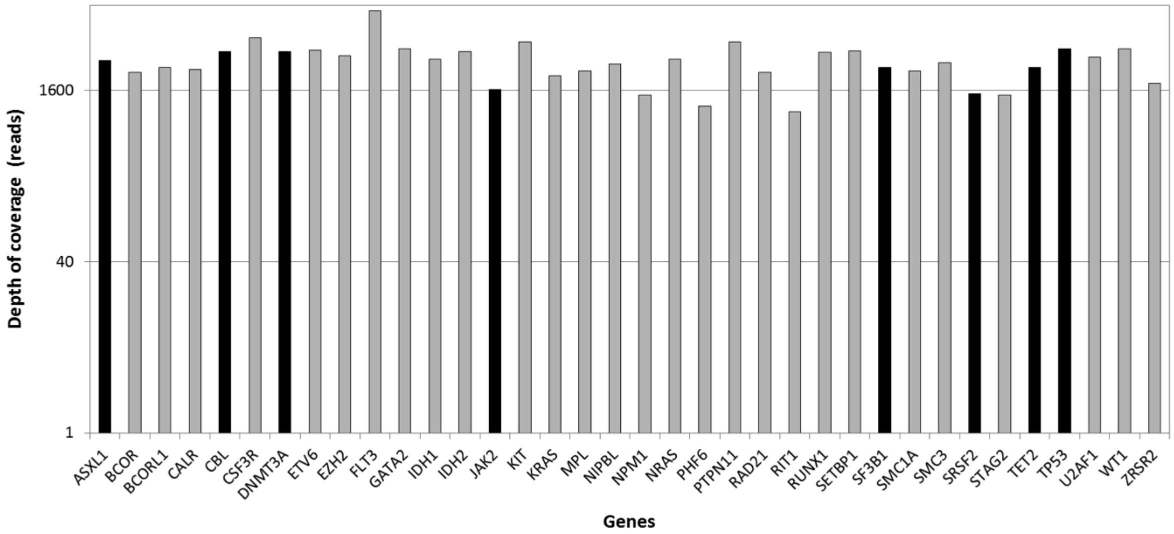

Figure S1: Median depth of sequencing coverage per gene. Black bars indicate genes that are frequently involved in clonal hematopoiesis.

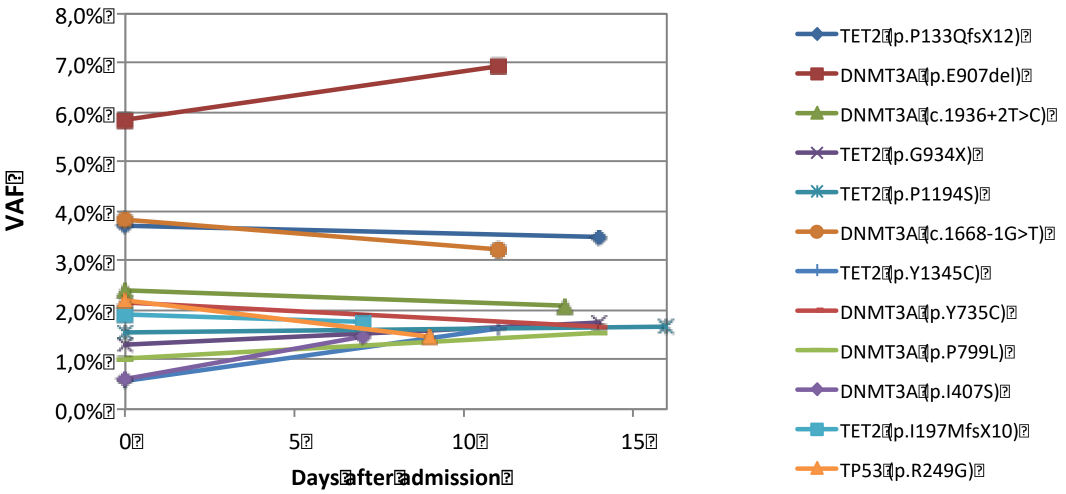

Figure S2: Repeated sequencing in 9 CH-positive patients.

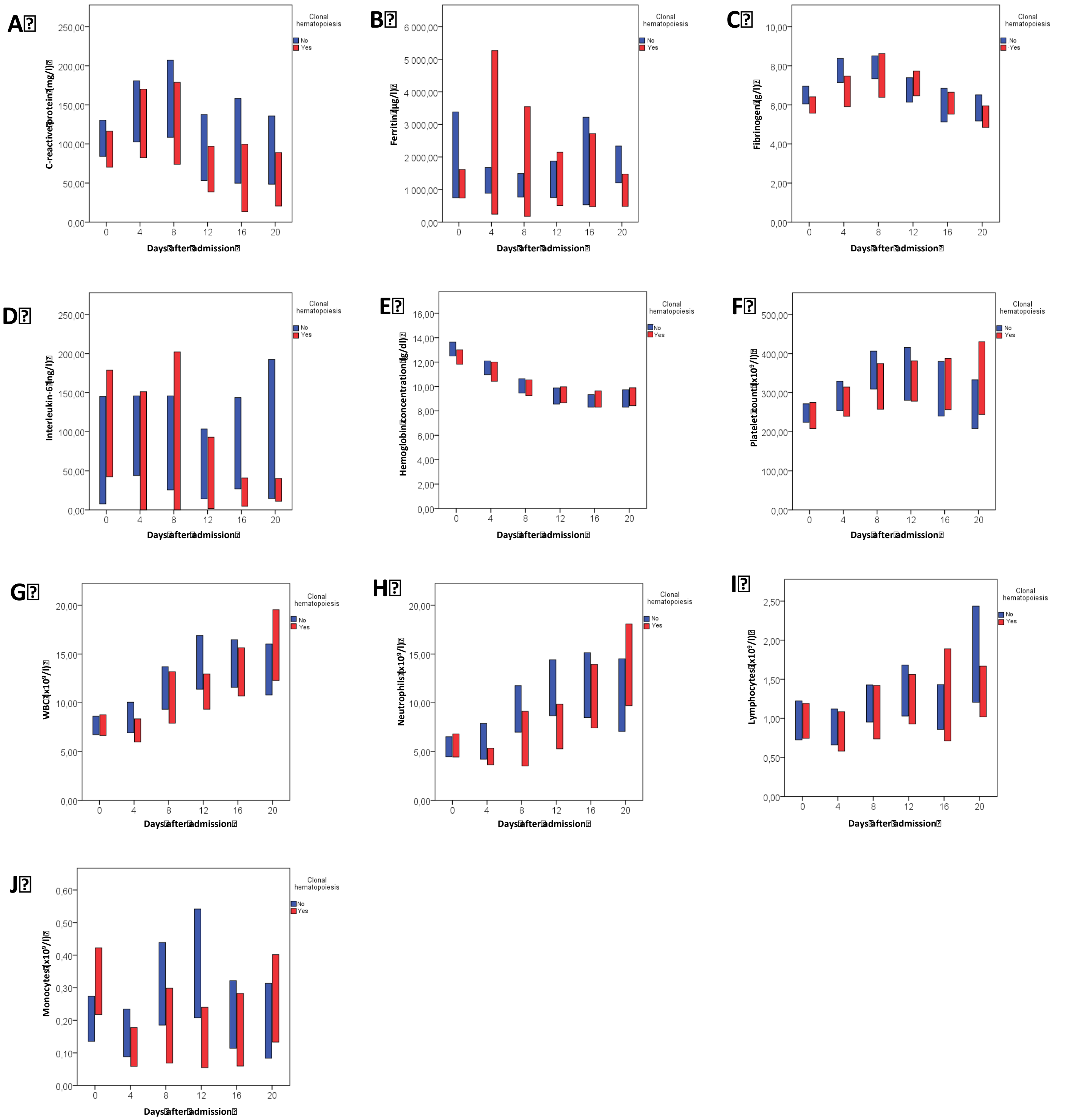

Figure S3: Kinetics of laboratory values during hospitalization in COVID-19 patients according to the presence (red boxes) or absence (blue boxes) of CH. Boxes indicate the mean values (95% CI) observed in patients at different timepoints (D0, D4, D8, D12, D16, D20). (**A**) C-reactive protein; (**B**) ferritin; (**C**) fibrinogen; (**D**) interleukin-6; (**E**) hemoglobin concentration; (**F**) platelet count; (**G**) WBC; (**H**) neutrophils; (**I**) lymphocytes; (**J**) monocytes.

Table S1: Determination of the threshold of detection of the high-throughput sequencing assay. These data were determined using multiplex NGS Tru-Q DNA 7 (Horizon Diagnostics) for seven point mutations in 65 independent experiments (performed under the same conditions as described for the present study).

Gene	Expected Variant	Expected VAF	Median VAF	Min	Max	Mean	SD	CV	Sensitivity
FLT3	c.2503G>T:p.D835Y	0,013	0,013	0,008	0,020	0,012	0,003	21%	97%
IDH1	c.395G>A:p.R132H	0,013	0,012	0,008	0,021	0,012	0,003	21%	92%
IDH2	c.419G>A:p.R140Q	0,013	0,014	0,008	0,024	0,014	0,003	22%	94%
IDH2	c.515G>A:p.R172K	0,013	0,012	0,008	0,026	0,013	0,003	26%	97%
JAK2	c.1849G>T:p.V617F	0,013	0,014	0,009	0,025	0,014	0,003	24%	95%
KRAS	c.183A>C:p.Q61H	0,013	0,014	0,008	0,024	0,014	0,003	23%	95%
NRAS	c.181C>A:p.Q61K	0,013	0,013	0,008	0,019	0,013	0,003	21%	92%

Table S2. Target genes and driver classification.

Gene	Transcript	Chromosome	Sequenced Exons	Criteria For Classification of Somatic Mutations as Driver
ASXL1	NM_015338	chr20	E11 and E12	Frameshift/nonsense/splice-site in exon 11–12
BCOR	NM_001123385	chrX	E02 to E15	Frameshift/nonsense/splice-site
BCORL1	NM_021946	chrX	E01 to E12	Frameshift/nonsense/splice-site
CALR	NM_004343	chr19	E09	Frameshift in exon 9
CBL	NM_005188	chr11	E08 and E09	Missense in Linker/RING finger domains (aa 366 to 427)
CSF3R	NM_156039	chr1	E14 to E18	Missense at T615 / T618 / T640; Frameshift/nonsense/splice-site in cytoplasmic domain (aa 681 to 863)
DNMT3A	NM_022552	chr2	E02 to E23	Frameshift/nonsense/splice-site; Missense (especially but not exclusively in Mtase domain (aa 632 to 795))
ETV6	NM_001987	chr12	E01 to E08	Frameshift/nonsense/splice-site; Missense in ETS domain (aa 338 to 424)
EZH2	NM_004456	chr7	E02 to E20	Frameshift/nonsense/splice-site; Missense in SET domain (aa 617 to 738)
FLT3	NM_004119	chr13	E20	Missense/inframe indel involving aa 835 to 842
GATA2	NM_032638	chr3	E02 to E06	Frameshift/nonsense/splice-site; Missense in ZF2 domain (aa 294 to 344 and 349 to 398)
IDH1	NM_005896	chr2	E04	Missense at R132
IDH2	NM_002168	chr15	E04	Missense at R140 / R172
JAK2	NM_004972	chr9	E12 and E14	Missense V617F; Missense/indel in aa 505 to 547
KIT	NM_000222	chr4	E08 to E11 and E17	Missense at D816 / N822; inframe indel involving D419
KRAS	NM_033360	chr12	E02 and E03	Missense at G12 / G13 / Q61
MPL	NM_005373	chr1	E10	Missense at S505 / W515
NIPBL	NM_133433	chr5	E02 to E47	Frameshift/nonsense/splice-site
NPM1	NM_002520	chr5	E11	Frameshift in exon 12 (4-bp insertions)
NRAS	NM_002524	chr1	E02 and E03	Missense at G12 / G13 / Q61
PHF6	NM_001015877	chrX	E02 to E11	Frameshift/nonsense/splice-site
PTPN11	NM_002834	chr12	E03 and E13	Missense involving aa 58 to 76 and aa 491 to 510
RAD21	NM_006265	chr8	E02 to E14	Frameshift/nonsense/splice-site
RIT1	NM_006912	chr1	E05	Missense involving aa 80 to 90
RUNX1	NM_001001890	chr21	E01 to E06	Frameshift/nonsense/splice-site; Missense in RUNT domain (aa 50 to 177)
SETBP1	NM_015559	chr18	E04	Missense involving aa 867 to 874
SF3B1	NM_012433	chr2	E13 to E16	Missense in terminal HEAT domains(aa 574-789)
SMC1A	NM_006306	chrX	E01 to E25	Frameshift/nonsense/splice-site; Missense at R96 / R586
SMC3	NM_005445	chr10	E01 to E29	Frameshift/nonsense/splice-site
SRSF2	NM_003016	chr17	E01	Missense/in frame deletion involving P95
STAG2	NM_001042749	chrX	E03 to E35	Frameshift/nonsense/splice-site
TET2	NM_001127208	chr4	E03 to E11	Frameshift/nonsense/splice-site; Missense in conserved domains (aa 1134 to 1444 and aa 1842 to 1921)
TP53	NM_001126112	chr17	E01 to E11	Frameshift/nonsense/splice-site; Missense in DNA-binding domain (aa 95 to 289)
U2AF1	NM_006758	chr21	E02 and E06	Missense at S34 / R156 / Q157
WT1	NM_024426	chr11	E07 and E09	Frameshift/nonsense/splice-site
ZRSR2	NM_005089	chrX	E01 to E11	Frameshift/nonsense/splice-site

Table S3. Variant calling of identified variants in other DNA samples sequenced in the same library (40 DNA samples in each library) and other libraries (4240 DNA samples with the same panel). Counts are generated with the Diagnostic Variants Database (DVD, CHU Lille).

Gene	Chromosome	Position (hg19)	Ref	Alt	Library ID	Counts in the Library	Counts in ll Librairies
ASXL1	chr20	31 021 374	C	CC	Lib1	1/40	1/4240
PTPN11	chr12	112 926 888	G	A	Lib1	1/40	8/4240
DNMT3A	chr2	25 457 165	ACTC	A	Lib1	1/40	1/4240
DNMT3A	chr2	25 466 765	A	G	Lib1	1/40	1/4240
TET2	chr4	106 157 899	G	T	Lib1	1/40	1/4240
DNMT3A	chr2	25 467 209	T	C	Lib1	1/40	2/4240
DNMT3A	chr2	25 467 470	AG	A	Lib1	1/40	2/4240
DNMT3A	chr2	25 457 243	G	T	Lib1	1/40	9/4240
TET2	chr4	106 196 520	A	AA	Lib1	1/40	1/4240
TET2	chr4	106 190 798	G	A	Lib1	1/40	3/4240
PHF6	chrX	133 551 331	A	G	Lib1	1/40	2/4240
DNMT3A	chr2	25 462 023	CAGAAGT	C	Lib2	1/40	1/4240
DNMT3A	chr2	25 463 536	C	T	Lib2	1/40	3/4240
ASXL1	chr20	31 022 433	GCCATCGGAGGGG GG	G	Lib2	1/40	1/4240
TET2	chr4	106 164 070	C	T	Lib2	1/40	1/4240
DNMT3A	chr2	25 467 208	C	A	Lib2	1/40	2/4240
TET2	chr4	106 182 995	A	G	Lib2	1/40	3/4240
DNMT3A	chr2	25 470 011	A	G	Lib2	1/40	2/4240
TET2	chr4	106 155 496	CC	C	Lib1	1/40	1/4240
DNMT3A	chr2	25 463 289	T	C	Lib3	2/40	17/4240
TET2	chr4	106 193 931	C	T	Lib1	1/40	10/4240
TP53	chr17	7 577 536	T	C	Lib1	1/40	1/4240
DNMT3A	chr2	25 463 524	G	A	Lib1	1/40	3/4240
DNMT3A	chr2	25 466 800	G	A	Lib1	1/40	17/4240
TP53	chr17	7 578 443	A	G	Lib1	1/40	1/4240
DNMT3A	chr2	25 463 289	T	C	Lib2	2/40	17/4240
DNMT3A	chr2	25 462 011	G	A	Lib2	1/40	1/4240
TET2	chr4	106 156 017	ACTAGCTGCAATGC TAA	A	Lib2	1/40	1/4240
DNMT3A	chr2	25 462 005	A	T	Lib2	1/40	1/4240
DNMT3A	chr2	25 469 548	A	C	Lib2	1/40	1/4240
TET2	chr4	106 155 689	TT	T	Lib2	1/40	1/4240
GATA2	chr3	128 202 850	T	G	Lib2	1/40	1/4240
SMC1A	chrX	53 410 128	T	G	Lib2	1/40	1/4240
TET2	chr4	106 156 747	C	T	Lib2	1/40	23/4240
ASXL1	chr20	31 022 364	A	TAA T	Lib2	1/40	1/4240
TET2	chr4	106 157 572	TC	T	Lib2	1/40	4/4240
TET2	chr4	106 163 998	C	CTC	Lib2	1/40	1/4240
CBL	chr11	119 149 260	T	G	Lib2	1/40	3/4240
DNMT3A	chr2	25 467 180	GA	GTG CGT	Lib2	1/40	1/4240
DNMT3A	chr2	25 463 289	T	C	Lib2	2/40	17/4240
TET2	chr4	106 157 002	C	T	Lib2	1/40	1/4240
DNMT3A	chr2	25 470 905	C	T	Lib2	1/40	1/4240
DNMT3A	chr2	25 466 797	C	A	Lib2	1/40	3/4240
U2AF1	chr21	44 514 777	T	C	Lib2	1/40	31/4240
TET2	chr4	106 196 839	T	G	Lib2	1/40	1/4240
DNMT3A	chr2	25 469 542	C	T	Lib2	1/40	3/4240

DNMT3A	chr2	25 463 212	T	C	Lib2	1/40	38/4240
TET2	chr4	106 180 925	A	G	Lib2	1/40	1/4240
DNMT3A	chr2	25 470 030	G	C	Lib3	1/40	2/4240
DNMT3A	chr2	25 457 242	C	T	Lib3	2/40	129/4240
TET2	chr4	106 157 375	CT	C	Lib3	1/40	1/4240
ASXL1	chr20	31 021 211	C	T	Lib3	1/40	8/4240
TP53	chr17	7 578 535	T	C	Lib3	1/40	4/4240
CBL	chr11	119 148 928	T	A	Lib3	1/40	2/4240
DNMT3A	chr2	25 464 475	T	A	Lib3	1/40	2/4240
DNMT3A	chr2	25 468 888	C	T	Lib3	1/40	4/4240
ASXL1	chr20	31 022 937	CC	C	Lib3	1/40	5/4240
IDH2	chr15	90 631 934	C	T	Lib3	1/40	286/4240
SRSF2	chr17	74 732 959	G	T	Lib3	2/40	187/4240
TET2	chr4	106 190 860	C	A	Lib3	1/40	1/4240
TP53	chr17	7 577 575	A	G	Lib3	1/40	2/4240
TET2	chr4	106 157 326	C	T	Lib3	1/40	1/4240
TET2	chr4	106 197 337	TAGGAAT	T	Lib3	1/40	1/4240
ASXL1	chr20	31 022 414	TAGAGAGGCGGCC ACCACTGCCAT	T	Lib3	1/40	126/4240
CBL	chr11	119 148 931	G	A	Lib3	1/40	7/4240
STAG2	chrX	123 199 798	TAAGT	T	Lib3	1/40	3/4240
TET2	chr4	106 156 707	AG	A	Lib3	1/40	1/4240
SF3B1	chr2	198 267 397	T	C	Lib3	1/40	4/4240
TET2	chr4	106 196 799	TTAGACCAAATGTA CATCAT	T	Lib3	1/40	1/4240
DNMT3A	chr2	25 466 825	GA	G	Lib3	1/40	7/4240
TET2	chr4	106 197 317	A	G	Lib3	1/40	3/4240
DNMT3A	chr2	25 469 530	C	G	Lib3	1/40	4/4240
BCORL1	chrX	129 147 923	C	T	Lib3	1/40	1/4240
TET2	chr4	106 156 747	C	T	Lib3	1/40	23/4240
TET2	chr4	106 164 794	G	C	Lib3	1/40	2/4240
DNMT3A	chr2	25 458 629	A	T	Lib3	1/40	28/4240
ASXL1	chr20	31 021 283	C	T	Lib3	1/40	2/4240
TET2	chr4	106 155 394	AGTGAA	A	Lib3	1/40	1/4240
DNMT3A	chr2	25 457 242	C	T	Lib3	2/40	129/4240
SRSF2	chr17	74 732 959	G	T	Lib3	2/40	187/4240
TET2	chr4	106 164 914	G	C	Lib3	1/40	1/4240
TET2	chr4	106 155 466	CG	C	Lib3	1/40	2/4240
DNMT3A	chr2	25 464 463	C	T	Lib3	1/40	1/4240
DNMT3A	chr2	25 463 289	T	C	Lib3	2/40	17/4240
TET2	chr4	106 156 348	C	T	Lib3	1/40	6/4240
TET2	chr4	106 196 491	T	G	Lib3	1/40	2/4240
TP53	chr17	7 578 413	C	T	Lib3	1/40	9/4240
DNMT3A	chr2	25 463 228	A	C	Lib3	1/40	2/4240
JAK2	chr9	5 073 770	G	T	Lib3	1/40	381/4240
TET2	chr4	106 158 442	CC	C	Lib3	1/40	2/4240
TET2	chr4	106 158 483	T	TT	Lib3	1/40	2/4240

Table S4. Clonal hematopoiesis-associated variants identified in hospitalized COVID-19-positive patients (*n* = 122).

UPN	Gene	Transcript	Type	Mutation	Mutared Reads	Total Reads	VAF	SIFT	PPH2	HSF
6	*ASXL1*	NM_015338	Frame_Shift_Ins	exon 11 c.1373dup : p.A459SfsX26	43	2294	1.9%	NA	NA	NA
6	*PTPN11*	NM_002834	Missense_Mutation	exon 13 c.1508G>A : p.G503E	219	8200	2.7%	Damaging	probably_damaging	NA
11	*DNMT3A*	NM_022552	In_Frame_Del	exon 23 c.2719_2721del : p.E907del	96	1381	7.0%	NA	NA	NA
13	*DNMT3A*	NM_022552	Splice_Site_Mutation	exon 16 c.1936+2T>C	80	3822	2.1%	NA	NA	Broken WT Donor Site
15	*TET2*	NM_001127208	Nonsense_Mutation	exon 3 c.2800G>T : p.G934X	67	3795	1.8%	NA	NA	NA
16	*DNMT3A*	NM_022552	Splice_Site_Mutation	exon 14 c.1668-2A>G	47	2772	1.7%	NA	NA	Broken WT Acceptor Site
16	*DNMT3A*	NM_022552	Frame_Shift_Del	exon 14 c.1605del : p.Y536TfsX115	38	2076	1.8%	NA	NA	NA
16	*DNMT3A*	NM_022552	Missense_Mutation	exon 23 c.2644C>A : p.R882S	32	1904	1.7%	Damaging	probably_damaging	NA
16	*TET2*	NM_001127208	Frame_Shift_Ins	exon 11 c.4853dup : p.Y1618X	36	1837	2.0%	NA	NA	NA
17	*TET2*	NM_001127208	Missense_Mutation	exon 9 c.4076G>A : p.R1359H	78	5056	1.5%	Damaging	probably_damaging	NA
18	*PHF6*	NM_001015877	Missense_Mutation	exon 9 c.967A>G : p.K323E	98	1534	6.4%	Damaging	possibly_damaging	NA
21	*DNMT3A*	NM_022552	In_Frame_Del	exon 20 c.2378_2383del : p.Y793_F794del	78	2965	2.6%	NA	NA	NA
24	*DNMT3A*	NM_022552	Missense_Mutation	exon 18 c.2146G>A : p.V716I	128	4167	3.1%	Damaging	benign	NA
25	*ASXL1*	NM_015338	Frame_Shift_Del	exon 12 c.1919_1932del : p.A640GfsX13	36	1783	2.0%	NA	NA	NA
26	*TET2*	NM_001127208	Missense_Mutation	exon 5 c.3580C>T : p.P1194S	20	1190	1.7%	Damaging	probably_damaging	NA
28	*DNMT3A*	NM_022552	Splice_Site_Mutation	exon 14 c.1668-1G>T	103	3186	3.2%	NA	NA	Broken WT Acceptor Site
28	*TET2*	NM_001127208	Missense_Mutation	exon 8 c.4034A>G : p.Y1345C	39	2371	1.6%	Damaging	probably_damaging	NA
35	*DNMT3A*	NM_022552	Missense_Mutation	exon 9 c.1031T>C : p.L344P	349	6530	5.3%	Damaging	probably_damaging	NA
36	*TET2*	NM_001127208	Frame_Shift_Del	exon 3 c.398del : p.P133QfsX12	138	3961	3.5%	NA	NA	NA
37	*DNMT3A*	NM_022552	Missense_Mutation	exon 19 c.2204A>G : p.Y735C	46	3115	1.5%	Tolerated	probably_damaging	NA
41	*TET2*	NM_001127208	Nonsense_Mutation	exon 10 c.4393C>T : p.R1465X	37	2483	1.5%	NA	NA	NA
47	*TP53*	NM_001126112	Missense_Mutation	exon 7 c.745A>G : p.R249G	36	2478	1.5%	Damaging	probably_damaging	NA
51	*DNMT3A*	NM_022552	Missense_Mutation	exon 18 c.2158C>T : p.R720C	106	6261	1.7%	Damaging	probably_damaging	NA
53	*DNMT3A*	NM_022552	Missense_Mutation	exon 16 c.1903C>T : p.R635W	756	2346	32.2%	Damaging	probably_damaging	NA
54	*TP53*	NM_001126112	Missense_Mutation	exon 5 c.487T>C : p.Y163H	50	3244	1.5%	Damaging	probably_damaging	NA
55	*DNMT3A*	NM_022552	Missense_Mutation	exon 19 c.2204A>G : p.Y735C	63	3802	1.7%	Tolerated	probably_damaging	NA
55	*DNMT3A*	NM_022552	Missense_Mutation	exon 20 c.2396C>T : p.P799L	30	1949	1.5%	Damaging	probably_damaging	NA
56	*TET2*	NM_001127208	Frame_Shift_Del	exon 3 c.919_934del : p.L307IfsX35	156	5552	2.8%	NA	NA	NA

59	*DNMT3A*	NM_022552	Missense_Mutation	exon 20 c.2402T>A : p.M801K	113	2432	4.6%	Damaging	probably_damaging	NA
62	*DNMT3A*	NM_022552	Missense_Mutation	exon 10 c.1220T>G : p.I407S	37	2532	1.5%	Damaging	probably_damaging	NA
62	*TET2*	NM_001127208	Frame_Shift_Del	exon 3 c.591del : p.I197MfsX10	60	3394	1.8%	NA	NA	NA
68	*GATA2*	NM_032638	Splice_Site_Mutation	exon 3 c.872-2A>C	175	2971	5.9%	NA	NA	Broken WT Acceptor Site
68	*SMC1A*	NM_006306	Missense_Mutation	exon 20 c.3020A>C : p.Q1007P	35	2394	1.5%	Tolerated	benign	NA
68	*TET2*	NM_001127208	Nonsense_Mutation	exon 3 c.1648C>T : p.R550X	41	2713	1.5%	NA	NA	NA
70	*ASXL1*	NM_015338	Nonsense_Mutation	exon 12 c.1849delinsTAAT : p.I617delinsX	722	7232	10.0%	NA	NA	NA
70	*TET2*	NM_001127208	Frame_Shift_Del	exon 3 c.2474del : p.S825X	432	3583	12.1%	NA	NA	NA
70	*TET2*	NM_001127208	Frame_Shift_Ins	exon 5 c.3508_3509insTC : p.Q1170LfsX57	203	1809	11.2%	NA	NA	NA
71	*CBL*	NM_005188	Missense_Mutation	exon 9 c.1268T>G : p.I423S	49	3212	1.5%	Damaging	probably_damaging	NA
74	*DNMT3A*	NM_022552	Frame_Shift_Ins	exon 15 c.1694delinsACGCA : p.L565HfsX14	208	3820	5.4%	NA	NA	NA
74	*DNMT3A*	NM_022552	Missense_Mutation	exon 19 c.2204A>G : p.Y735C	90	3882	2.3%	Tolerated	probably_damaging	NA
74	*TET2*	NM_001127208	Nonsense_Mutation	exon 3 c.1903C>T : p.Q635X	28	1831	1.5%	NA	NA	NA
76	*DNMT3A*	NM_022552	Splice_Site_Mutation	exon 7 c.855+1G>A	276	1138	24.3%	NA	NA	Broken WT Donor Site
78	*DNMT3A*	NM_022552	Missense_Mutation	exon 16 c.1906G>T : p.V636L	40	2691	1.5%	Damaging	probably_damaging	NA
78	*U2AF1*	NM_006758	Missense_Mutation	exon 6 c.470A>G : p.Q157R	517	5321	9.7%	Damaging	probably_damaging	NA
80	*TET2*	NM_001127208	Nonsense_Mutation	exon 11 c.5172T>G : p.Y1724X	98	4866	2.0%	NA	NA	NA
81	*DNMT3A*	NM_022552	Nonsense_Mutation	exon 10 c.1226G>A : p.W409X	51	2890	1.8%	NA	NA	NA
82	*DNMT3A*	NM_022552	Missense_Mutation	exon 19 c.2281A>G : p.M761V	241	3593	6.7%	Damaging	probably_damaging	NA
82	*TET2*	NM_001127208	Missense_Mutation	exon 7 c.3953A>G : p.E1318G	40	2695	1.5%	Damaging	probably_damaging	NA
84	*DNMT3A*	NM_022552	Splice_Site_Mutation	exon 8 c.1015-3C>G	34	1704	2.0%	NA	NA	Broken WT Acceptor Site
84	*DNMT3A*	NM_022552	Missense_Mutation	exon 23 c.2645G>A : p.R882H	39	1690	2.3%	Damaging	possibly_damaging	NA
84	*TET2*	NM_001127208	Frame_Shift_Del	exon 3 c.2280del : p.P761LfsX52	79	1165	6.8%	NA	NA	NA
85	*ASXL1*	NM_015338	Nonsense_Mutation	exon 11 c.1210C>T : p.R404X	2389	7494	31.9%	NA	NA	NA
85	*TP53*	NM_001126112	Missense_Mutation	exon 5 c.395A>G : p.K132R	42	2819	1.5%	Damaging	probably_damaging	NA
87	*CBL*	NM_005188	Missense_Mutation	exon 8 c.1148T>A : p.I383K	20	944	2.1%	Damaging	probably_damaging	NA
87	*DNMT3A*	NM_022552	Nonsense_Mutation	exon 17 c.2038A>T : p.K680X	38	2552	1.5%	NA	NA	NA
88	*DNMT3A*	NM_022552	Splice_Site_Mutation	exon 12 c.1474+1G>A	41	2739	1.5%	NA	NA	Broken WT Donor Site
90	*ASXL1*	NM_015338	Frame_Shift_Del	exon 12 c.2423del : p.P808LfsX10	585	4496	13.0%	NA	NA	NA
90	*IDH2*	NM_002168	Missense_Mutation	exon 4 c.419G>A : p.R140Q	25	1703	1.5%	Damaging	probably_damaging	NA

90	*SRSF2*	NM_003016	Missense_Mutation	exon 1 c.284C>A : p.P95H	70	707	9.9%	Damaging	probably_damaging	NA
90	*TET2*	NM_001127208	Missense_Mutation	exon 9 c.4138C>A : p.H1380N	125	3882	3.2%	Damaging	probably_damaging	NA
90	*TP53*	NM_001126112	Missense_Mutation	exon 7 c.706T>C : p.Y236H	26	1710	1.5%	Damaging	probably_damaging	NA
91	*TET2*	NM_001127208	Nonsense_Mutation	exon 3 c.2227C>T : p.Q743X	40	2741	1.5%	NA	NA	NA
91	*TET2*	NM_001127208	In_Frame_Del	exon 11 c.5671_5676del : p.R1891_N1892del	39	2641	1.5%	NA	NA	NA
92	*ASXL1*	NM_015338	Frame_Shift_Del	exon 12 c.1900_1922del : p.E635RfsX15	32	812	3.9%	NA	NA	NA
92	*CBL*	NM_005188	Missense_Mutation	exon 8 c.1151G>A : p.C384Y	45	1467	3.1%	Damaging	probably_damaging	NA
93	*STAG2*	NM_001042749	Splice_Site_Mutation	exon 21 c.2096+3_2096+6del	38	1374	2.8%	NA	NA	Broken WT Donor Site
95	*TET2*	NM_001127208	Frame_Shift_Del	exon 3 c.1609del : p.E537SfsX5	21	1127	1.9%	NA	NA	NA
99	*SF3B1*	NM_012433	Missense_Mutation	exon 14 c.1960A>G : p.S654G	33	2235	1.5%	Damaging	probably_damaging	NA
99	*TET2*	NM_001127208	Frame_Shift_Del	exon 11 c.5133_5151del : p.I1711MfsX2	1878	5672	33.1%	NA	NA	NA
101	*DNMT3A*	NM_022552	Frame_Shift_Del	exon 16 c.1877del : p.V626AfsX25	37	2551	1.5%	NA	NA	NA
101	*TET2*	NM_001127208	Missense_Mutation	exon 11 c.5650A>G : p.T1884A	40	2736	1.5%	Damaging	probably_damaging	NA
102	*DNMT3A*	NM_022552	Missense_Mutation	exon 10 c.1238G>C : p.G413A	54	3587	1.5%	Damaging	probably_damaging	NA
103	*BCORL1*	NM_021946	Missense_Mutation	exon 3 c.1175C>T : p.P392L	48	1596	3.0%	Damaging	benign	NA
104	*TET2*	NM_001127208	Nonsense_Mutation	exon 3 c.1648C>T : p.R550X	44	2828	1.6%	NA	NA	NA
104	*TET2*	NM_001127208	Missense_Mutation	exon 6 c.3662G>C : p.C1221S	41	2747	1.5%	Damaging	probably_damaging	NA
105	*DNMT3A*	NM_022552	Missense_Mutation	exon 22 c.2544T>A : p.F848L	38	2312	1.6%	Tolerated	benign	NA
107	*ASXL1*	NM_015338	Nonsense_Mutation	exon 11 c.1282C>T : p.Q428X	110	5491	2.0%	NA	NA	NA
107	*TET2*	NM_001127208	Frame_Shift_Del	exon 3 c.296_300del : p.S99TfsX19	40	2722	1.5%	NA	NA	NA
108	*DNMT3A*	NM_022552	Missense_Mutation	exon 23 c.2645G>A : p.R882H	603	1528	39.5%	Damaging	possibly_damaging	NA
108	*SRSF2*	NM_003016	Missense_Mutation	exon 1 c.284C>A : p.P95H	23	643	3.6%	Damaging	probably_damaging	NA
108	*TET2*	NM_001127208	Missense_Mutation	exon 6 c.3782G>C : p.R1261P	1573	11346	13.9%	Damaging	probably_damaging	NA
110	*TET2*	NM_001127208	Frame_Shift_Del	exon 3 c.368del : p.R123LfsX5	39	2612	1.5%	NA	NA	NA
117	*DNMT3A*	NM_022552	Missense_Mutation	exon 17 c.2050G>A : p.V684I	113	1277	8.8%	Tolerated	possibly_damaging	NA
119	*DNMT3A*	NM_022552	Missense_Mutation	exon 19 c.2204A>G : p.Y735C	36	2353	1.5%	Tolerated	probably_damaging	NA
119	*TET2*	NM_001127208	Nonsense_Mutation	exon 3 c.1249C>T : p.Q417X	119	2462	4.8%	NA	NA	NA
119	*TET2*	NM_001127208	Nonsense_Mutation	exon 11 c.4824T>G : p.Y1608X	310	1700	18.2%	NA	NA	NA
120	*TP53*	NM_001126112	Missense_Mutation	exon 5 c.517G>A : p.V173M	75	3570	2.1%	Damaging	probably_damaging	NA
121	*DNMT3A*	NM_022552	Missense_Mutation	exon 19 c.2265T>G : p.F755L	32	2075	1.5%	Damaging	probably_damaging	NA
121	*JAK2*	NM_004972	Missense_Mutation	exon 14 c.1849G>T : p.V617F	378	2695	14.0%	Damaging	probably_damaging	NA
121	*TET2*	NM_001127208	Frame_Shift_Del	exon 3 c.3344del : p.P1115LfsX2	146	2293	6.4%	NA	NA	NA
121	*TET2*	NM_001127208	Frame_Shift_Ins	exon 3 c.3384dup : p.D1129X	60	1705	3.5%	NA	NA	NA

UPN, unit patient number; NA, not applicable; PPH2, polyphen-2; HSF, human splicing finder.

Table S5. Frequency of clonal hematopoiesis, DNMT3A mutations and TET2 mutations among age groups in the retrospective cohort according to gender.

Mutations	Age Groups	Females	Males	Total	*p*-Value
Clonal hematopoiesis	All	56 (29%)	48 (26%)	104 (28%)	0.490
	<60 y	2 (4%)	6 (16%)	8 (10%)	
	60–70 y	12 (23%)	11 (20%)	23 (21%)	
	70–80 y	26 (41%)	22 (33%)	48 (37%)	
	>80 y	16 (53%)	9 (33%)	25 (44%)	
DNMT3A	All	37 (19%)	30 (16%)	67 (18%)	0.501
	<60 y	1 (2%)	6 (16%)	7 (9%)	
	60–70 y	12 (23%)	8 (15%)	20 (19%)	
	70–80 y	16 (25%)	11 (17%)	27 (21%)	
	>80 y	8 (27%)	5 (19%)	13 (23%)	
TET2	All	15 (8%)	16 (9%)	31 (8%)	0.852
	<60 y	0 (0%)	1 (3%)	1 (1%)	
	60–70 y	0 (0%)	2 (4%)	2 (2%)	
	70–80 y	8 (13%)	10 (15%)	18 (14%)	
	>80 y	7 (23%)	3 (11%)	10 (18%)	
